# Integrated Controller Design and Application for CNC Machine Tool Servo Systems Based on Model Reference Adaptive Control and Adaptive Sliding Mode Control

**DOI:** 10.3390/s23249755

**Published:** 2023-12-11

**Authors:** Taihao Zhang, Xuewei Li, Hongdong Gai, Yuheng Zhu, Xiang Cheng

**Affiliations:** 1 School of Mechanical Engineering, Shandong University of Technology, Zibo 255000, China; 2 Qilu Division, Sinopec Catalyst Co., Ltd., Zibo 255000, China

**Keywords:** dynamics model for CNC machines, nonlinear friction, model reference adaptive control, adaptive sliding mode control

## Abstract

In order to reduce the effect of nonlinear friction and time-varying factors on the servo system of a computer numerical control (CNC) machine tool and improve its motion control accuracy, this paper uses an adaptive sliding mode control (ASMC) method based on model reference adaptive control (MRAC). The method adopts ASMC in the control outer loop and obtains the optimal control parameters by making the sliding mode control (SMC) law continuous and adaptively estimating the control parameters. At the same time, MRAC is used in the control inner loop to enhance the “invariance” of the controlled object so that the switching gain of SMC can satisfy the disturbance matching condition even under lesser conditions. Simulation and experimental results show that compared with the traditional SMC, the ASMC based on MRAC proposed in this paper effectively reduces the influence of nonlinear friction on the system performance, and the reduction in following error reaches 71.2%, which significantly improves the motion control accuracy of the control system. The spectral analysis of the following errors shows that the maximum magnitude reduction rate of the high-frequency chattering is 89.02%, which significantly reduces the effect of the high-frequency chattering and effectively improves the stability performance of the control system.

## 1. Introduction

The proportional integral derivative (PID) control strategy is widely employed in the servo control systems of CNC machine tools due to its stability, simplicity, and convenience [1]. However, in the actual machining process, the PID control strategy gradually fails to meet the requirement of motion control accuracy due to nonlinear friction and time-varying factors. As a typical nonlinear control method, SMC has gradually become a research hotspot in the field of automatic control due to its simple structure, ease of implementation, and robustness. Due to its discontinuous switching characteristics, it is prone to the chattering phenomenon, which affects the control performance of the system. Therefore, it is important to investigate a novel nonlinear SMC strategy to improve the motion accuracy of servo control systems.

In traditional SMC, the alternating positive and negative output signals of the sign function are the root cause of chatter. In order to mitigate the negative impact of chatter on system control performance, References [2,3,4] propose a new SMC method to reduce chatter generation. In addition, References [5,6] enhance the stability of the control system by designing a high-order sliding mode controller. In order to improve the robustness of the system, researchers choose SMC with integral sliding mode function methods [7,8,9,10] and disturbance estimation methods [11,12,13]. Reference [14] shows that SMC is a reliable and robust control method under the condition of disturbance matching. However, when the parameters of the model are uncertain, the dynamics are not modeled, or the external disturbance is mismatched, it is difficult to achieve synchronization between different model representations and systems of different orders, which affects the control performance of SMC.

Therefore, when designing a sliding mode controller, in order to satisfy the disturbance matching condition of SMC, it is necessary first to obtain an upper bound on the nonlinear disturbance of the controlled object. Moreover, the nonlinear disturbance upper bound is often easily overestimated, and even if the upper bound can be accurately estimated, if the nonlinear disturbance upper bound of the controlled object is too large, then the SMC needs stronger anti-disturbance capability to satisfy the disturbance matching condition, which still leads to intensely chattering in the control system, and it is difficult to achieve better control performance just by optimizing the SMC.

To address the effects caused by SMC, researchers have combined it with other control methods to optimize control performance. It includes adaptive sliding mode control [15,16], neural network sliding mode control [17,18,19] and fuzzy sliding mode control [20,21]. Among them, the development of adaptive control is relatively mature and can adapt to the state changes in the system by adjusting the characteristics of the control system. Combining SMC with adaptive control can effectively mitigate SMC the issue of chattering while improving the control performance of nonlinear systems.

In References [22,23,24], ASMC is designed under the condition that the upper limit of nonlinear disturbance is unknown so that the system can reach the convergence state in a short time. However, it is pointed out in Reference [24] that when the initial value of the adaptive gain parameter is underestimated, it is easy for the adaptive gain parameter to increase indefinitely. To address this issue, Reference [25] presented an adaptive sliding mode method that compensates for uncertain nonlinearities and linear uncertain parameters in the system. In addition, Reference [26] proposed a sensor robust tracking control strategy based on the ASMC method to ensure the stability of the system. Reference [27] developed an adaptive sliding mode control with a fractional-order model predictive control algorithm to accurately follow a given trajectory.

In summary, current adaptive control methods have two main approaches to reducing the chatter generated by SMC:

(1) Adaptive estimation of the unknown variables as known quantities that can be controlled directly.
(2) Adaptive estimation of the SMC parameters to obtain control parameters that can achieve optimal control performance.

Although the above control methods mitigate the effect of chattering in SMC and improve the system response speed, they do not start from the root cause of chattering in SMC to reduce the upper bound of uncertainty factors as much as possible, and the uncertainty factors of the object to be controlled are still large, which leads to the control algorithms to become more complex with the improvement of the robustness and depend on the uncertainty factors of upper bound.

In this paper, starting from the reduction in the upper bound of the uncertainty factor, the upper bound of the uncertainty factor is reduced to some extent by the model-independent nonlinear control method, which reduces the degree of uncertainty of the controlled object (this part of the work has been completed in Reference [28]). Then, the SMC is applied to radically reduce the effect of chattering, which is based on the adaptive estimation of the SMC parameters using adaptive algorithms to improve the control performance of the system further.

The main framework of the paper is as follows: In Section 2, a mechanical model of a CNC machine tool with nonlinear friction is introduced. In Section 3, a novel ASMC strategy based on MRAC is proposed. In Section 4 and Section 5, the effectiveness of proposed ASMC is verified through simulation and experimentation. The paper is summarized in Section 6.

## 2. Numerical Control Machine Tool Dynamics Model

### 2.1. Mechanical Transmission Model

**Figure 1:**
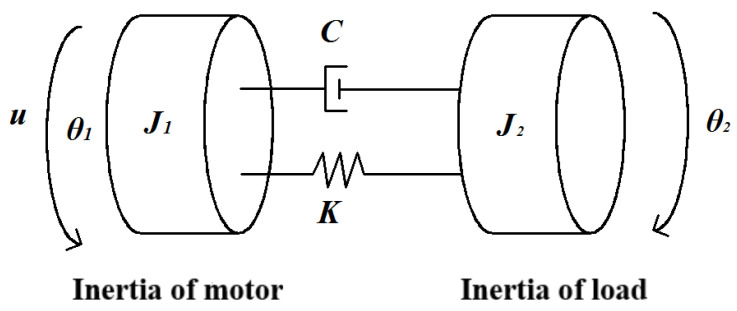
Equivalent double inertia mechanical model.

The mechanical transmission link of a CNC machine tool can be considered an “inertia-stiffness-damping” system. Due to the distinct characteristics of each component, accurate modeling can become overly complex if the components are treated as flexible links. To facilitate the study and analysis while accurately expressing the resonance properties, the mechanical transmission link is simplified in this paper to a double inertia model with only motor and load inertia, as illustrated in Figure 1.

In Figure 1, *u* is the torque output of the motor, *θ*_1_ and *θ*_2_ are the equivalent angular displacements of the motor and the load, respectively. The specific values are shown in Table 1. Based on the equivalent double inertia model, the kinetic differential equation is formulated as follows.

(1) $$u=J_{1}{\ddot{\theta }}_{1}+C\left({\dot{\theta }}_{1}-{\dot{\theta }}_{2}\right)+K\left(\theta_{1}-\theta_{2}\right)$$

(2) $$C\left({\dot{\theta }}_{1}-{\dot{\theta }}_{2}\right)+K\left(\theta_{1}-\theta_{2}\right)=J_{2}{\ddot{\theta }}_{2}$$

### 2.2. LuGre Friction Model

Nonlinear friction has a significant impact on the control performance of servo systems, which is mainly reflected in the phenomenon of “Zero crossing” and “Over-quadrant sharp corners” when the velocity changes direction. This paper involves the nonlinear friction model LuGre friction model [29]; the model effectively displays the static and dynamic features of nonlinear friction. Equation (3) demonstrates the correlation between friction and velocity.

(3) $$\left\{\begin{array}{c} s\left(V\right)=F_{c}+\left(F_{s}-F_{c}\right)e^{{-\left(\frac{V}{V_{s}}\right)}^{2}}\\ \dot{z}=v-\frac{\sigma_{0}z\left|V\right|}{s\left(V\right)}\\ F=\sigma_{0}z+\sigma_{1}z+\sigma_{2}V \end{array}\right.$$

**Figure 2:**
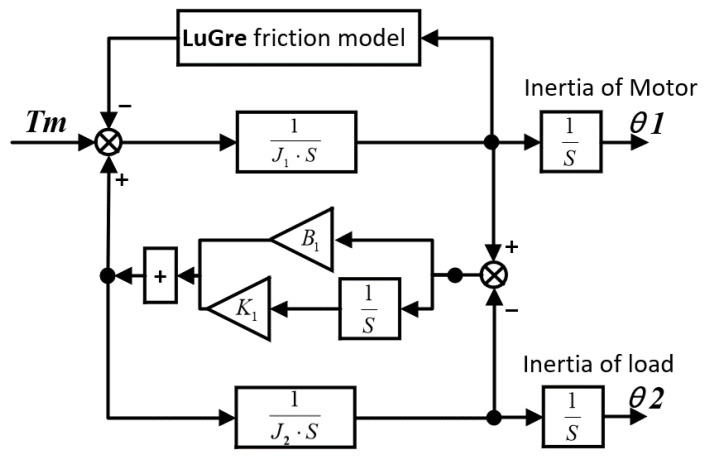
Simulink model of CNC machine tool dynamics.

Figure 2 shows the equivalent double inertia mechanical Simulink model, including LuGre friction, and the specific friction parameters are shown in Table 2.

## 3. Integrated Control Design of CNC Machine Tool Servo System Based on MRAC and ASMC

### 3.1. Structural Framework for ASMC Based on MRAC

**Figure 3:**
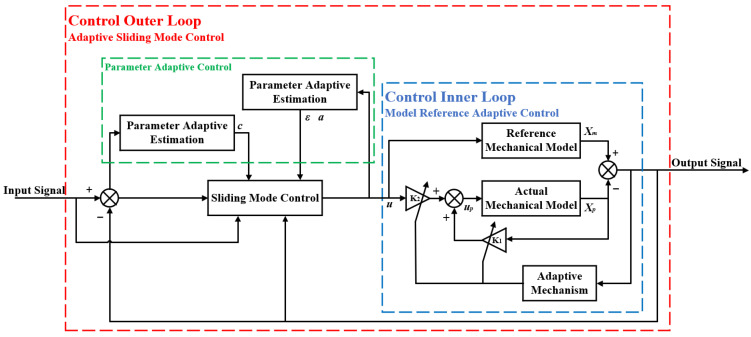
Structural framework for ASMC based on MRAC.

According to the research of the team [28], the results show that the MRAC effectively suppresses the influence of noise and mechanical resonance, enhances the precision of motion control and the resistance to interference in the control system, significantly enhances the “invariance” of the controlled object, and at the same time diminish the uncertainty of the nonlinear factors. In this study, MRAC is applied to the control inner loop, and based on this, a sliding mode controller and a parameter adaptive controller are designed to implement the control outer loop by ASMC to achieve the optimal parameter solution. The structural framework for ASMC based on MRAC is illustrated in Figure 3.

### 3.2. Sliding Mode Controller Design

Defining the following error of the CNC machine:

(4) $$e=r-\theta_{1}$$

where *r* is the expected input signal.

Defining the sliding surface:

(5) $$s=ce+\dot{e}$$

where *c* is the positive constant.

For SMC, since no precise, controlled model is needed in the theoretical design process, stiffness *K* and damping *C* are ignored as external disturbances and the single inertial model is simplified for the convenience of design.

(6) $$u=J{\ddot{\theta }}_{1}$$

where the total inertia *J* = *J*_1_ + *J*_2_.

Usually, CNC machine tools do not involve step signal control of larger amplitudes during operation, so the convergence law of the SMC adopts a relatively simple equal-velocity convergence law:

(7) $$\dot{s}=-\varepsilon \;sgn\left(s\right)$$

where *ε* is the switching gain and is the positive constant.

By combining Equations (4)–(7),obtain the SMC law as follows:

(8) $$u=J\left[\ddot{r}+c\dot{e}+\varepsilon \;sgn\left(s\right)\right]$$

In Equation (8), $J\left(\ddot{r}+c\dot{e}\right)$ is the equivalence term, also known as the linear term, which is able to achieve full control of the known linear characteristics of the model. $J\varepsilon \;sgn\left(s\right)$ is the switching term, also known as the nonlinear term; the project realizes the control of unknown nonlinear factors in the form of high-frequency chattering, and the control strength of the switching term needs to be greater than the maximum strength of the disturbance of the nonlinear factors, which is to satisfy the condition of disturbance matching.

For the proposed simplified system model (6) and control law (8), it is necessary to prove the stability of the control algorithm to ensure the stability of the system.

Define the *V* function:

(9) $$V\left(s\right)=s^{2}$$

Take the derivative of Equation (9) in time and then substitute it into Equation (7):

(10) $$\dot{V}=2s\dot{s}=-2s\varepsilon \;sgn\left(s\right)=-2\varepsilon \left|s\right|\leq 0$$

Obviously, Equation (9) is positive definite, and Equation (10) is negative definite; both satisfy the stability conditions of Lyapunov’s stability theory, so ${lim}_{t\to +\infty }s=0$, the system is uniformly asymptotically stable at the origin, and the state variable associated with the following error when s→0: $e\to 0,\dot{e}\to 0$.

### 3.3. Design of Sliding Mode Controller Based on Parametric Adaptive Control

In the SMC law (8), there is a discontinuous sign function (sign function), which causes discontinuity in the output signal and thus leads to chatter in the SMC. The use of a continuously derivable bipolar function (sigmoid function [30]) instead of the sign function can make the output signal relatively smooth, and the bipolar function is defined as follows [31]:

(11) $$\Phi \left(s\right)=\frac{2}{1+e^{-as}}-1$$

**Figure 4:**
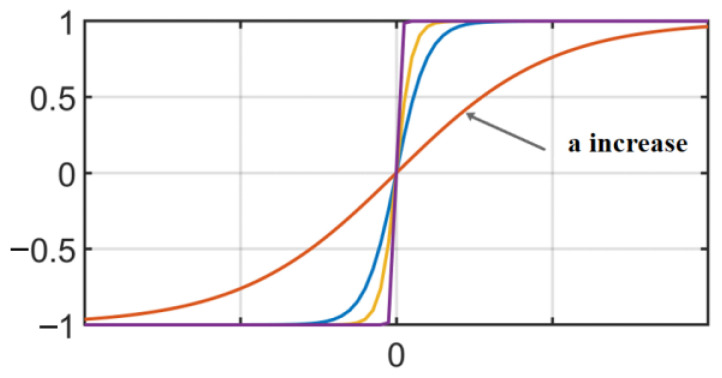
Bipolar function schematic.

The bipolar function is shown schematically in Figure 4. As the boundary thickness *a* increases, the bipolar function tends to become more similar to the sign function.

Rewrite the SMC law as follows:

(12) $$u=J\left[c\dot{e}+\ddot{r}+\hat{\varepsilon }\left(\frac{2}{1+e^{-\hat{a}s}}-1\right)\right]$$

where $\hat{\varepsilon },\;\hat{a}$ are the control parameters that require adaptive estimation, and the parameter estimation errors are $\tilde{\varepsilon }=\hat{\varepsilon }-\varepsilon$, $\tilde{a}=\hat{a}-a$; *ε*, *a* are the values at the final steady state, which is also the value to achieve the optimal control performance. By using the method of adaptive estimation of control parameters, it is possible to obtain control parameters with good control performance without determining the upper bound of nonlinear disturbances.

Define the *V* function and find the time derivative:

(13) $$V\left(e\right)=0.5e^{2}$$

(14) $$\dot{V}=e\frac{\partial e}{\partial \theta_{1}}\frac{\partial \theta_{1}}{\partial u}\frac{\partial u}{\partial \hat{\varepsilon }}\frac{\partial \hat{\varepsilon }}{\partial t}+e\frac{\partial e}{\partial \theta_{1}}\frac{\partial \theta_{1}}{\partial u}\frac{\partial u}{\partial \hat{a}}\frac{\partial \hat{a}}{\partial t}$$

In Equation (14), *e* is the following error, the sampling frequency of the CNC machine tool *T*_0_ = 0.48 ms, which can be organized by using $\frac{∆\theta_{1}}{∆u}$ instead of $\frac{\partial \theta_{1}}{\partial u}$ to represent the discrete system:

(15) $$\dot{V}=-Je\frac{∆\theta_{1}}{∆u}\left[\varphi \left(s,\hat{a}\right)\right]\dot{\hat{\varepsilon }}-Je\frac{∆\theta_{1}}{∆u}\left[\hat{\varepsilon }\frac{2se^{-\hat{a}s}}{{\left(1+e^{-\hat{a}s}\right)}^{2}}\right]\dot{\hat{a}}$$

To ensure that $\dot{V}\leq 0$ and the equality sign holds only when *e* = 0 or *s* = 0, let the adaptive law be:

(16) $$\dot{\hat{\varepsilon }}=\gamma_{\varepsilon }e\;sgn\left(∆\theta_{1}\cdot ∆u\right)\varphi \left(s,\hat{a}\right)$$

(17) $$\dot{\hat{a}}=\gamma_{a}e\;sgn\left(∆\theta_{1}\cdot ∆u\right)s$$

Substituting Equations (16) and (17) into Equation (15) yields the following results:

(18) $$\dot{V}=-J\gamma_{\varepsilon }e^{2}\left|\frac{∆\theta_{1}}{∆u}\right|\left[\varphi^{2}\left(s,\hat{a}\right)\right]-J\gamma_{a}e^{2}\left|\frac{∆\theta_{1}}{∆u}\right|\left[\hat{\varepsilon }\frac{2s^{2}e^{-\hat{a}s}}{{\left(1+e^{-\hat{a}s}\right)}^{2}}\right]$$

In Equation (18), $\hat{\varepsilon }>0$, then $\dot{V\leq 0}$, the equation holds only when *e* = 0 or *s* = 0. Then $\dot{V}$ is negative definite, and according to the second approach of Lyapunov’s stability theory:

(1) $V\left(e\right)$ is positive definite;
(2) $\dot{V}\left(e\right)$ is negative definite.

Therefore, the system is located at the origin, and the following error at zero is asymptotically stable.

Thus far, the adaptive algorithm for the switching gain *ε* and boundary thickness *a* has been determined as follows:

(19) $$\hat{\varepsilon }=\gamma_{\varepsilon }\int e\;sgn\left(∆\theta_{1}\cdot ∆u\right)\varphi \left(s,\hat{a}\right)dt+k_{\varepsilon_{0}}$$

(20) $$\hat{a}=\gamma_{a}\int e\;sgn\left(∆\theta_{1}\cdot ∆u\right)sdt+k_{a_{0}}$$

where *k_ε_*_0_ and *k_a_*_0_ are the initial values of the adaptive estimation of each parameter.

## 4. Simulation Analysis and Sliding Mode Control Parameters Adaptive Optimization

Based on the control framework diagram shown in Figure 3, the Matlab/Simulink simulation model is established by combining the derived ASMC law, where the MRAC structure containing the LuGre friction model is used as the control inner loop, while the ASMC implements the control outer loop. The values of the relevant parameters for the preliminary simulation are *γ_ε_* = 800, *k_ε_*_0_ = 10, *γ_a_* = 10, *k_a_*_0_ = 5, *c* = 30; the input desired position signal is $r=-cos\left(0.5\pi t\right)+1\mathrm{mm}$; the simulation time is 12 s; there is a total of 3 cycles of the input signal.

**Figure 5:**
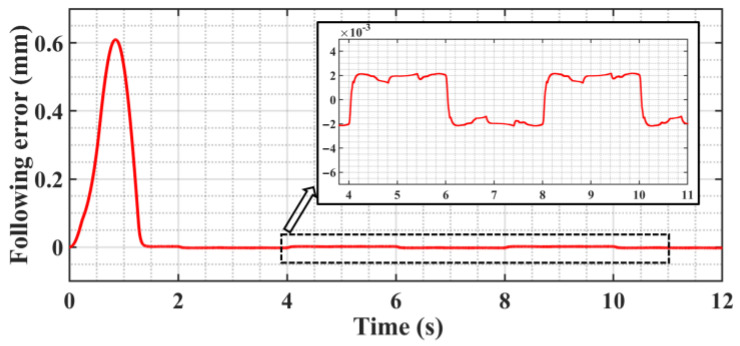
Trajectory following error curve.

**Figure 6:**
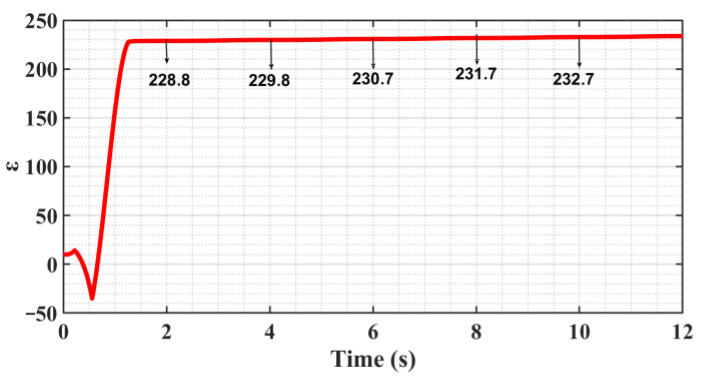
Switching gain *ε* change curve.

**Figure 7:**
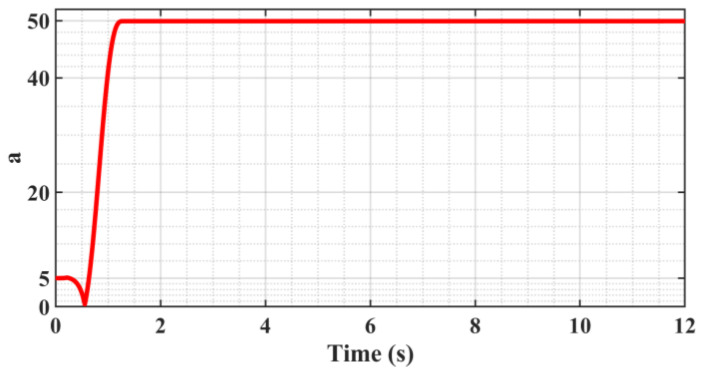
Boundary thickness *a* change curve.

Figure 5 shows the trajectory following the error curve; the system enters the steady state after 1.7 s after adaptive adjustment and produces a “static difference” of about 2 μm, and the “Static difference” direction is in alignment with the direction of motion velocity. And there is no “Zero crossing” or “Over-quadrant sharp corners” phenomenon during the velocity reversal change phase. Figure 6 shows that the presence of the “static difference” causes the switching gain *ε* to increase slowly, and Figure 7 shows that the value of the boundary thickness *a* stabilizes at 50.

From Figure 5, Figure 6 and Figure 7, on the one hand, it can be seen that the continuous slow increase in the switching gain *ε* does not make the following error appear to be further reduced because the control algorithm includes several adaptive algorithms, and the adaptive control itself has a certain time lag, which causes the convergence of the algorithm to be reduced in the case of time-varying signal input. On the other hand, it exacerbates the deficiency of convergence by the presence of sliding friction resistance, so increasing convergence can reduce the “static difference” to some extent.

It is known that the sliding surface is shown in Equation (5), for which the differential equation is solved to obtain:

(21) $$\left\{\begin{array}{c} e=e\left(0\right)e^{-ct}\\ \dot{e}=-ce\left(0\right)e^{-ct} \end{array}\right.$$

By analyzing the equation above, it becomes evident that the error amount decays exponentially to zero, and a positive correlation exists between the decay rate and the value of *c*. Therefore, appropriately increasing the value of *c* can enhance the convergence of the control algorithm.

The adaptive estimation of the parameter *c* is performed, and the parameter estimation error is defined:

(22) $$\tilde{c}=\hat{c}-c$$

Deriving the adaptive law based on Lyapunov stability theory, define the *V* function and derive it for time as follows: Equation (24) is obtained by putting Equations (4)–(6), (11) and (12) into the derivative of Equation (23):

(23) $$V=0.5\left(s^{2}+{\tilde{c}}^{2}\right)$$

(24) $$\dot{V}=s\dot{s}+\tilde{c}\dot{\hat{c}}=s\left(c\dot{e}+\ddot{r}-\frac{u}{J}\right)+\tilde{c}\dot{\hat{c}}=s\left[c\dot{e}-\hat{c}\dot{e}-\varepsilon \varphi \left(s\right)\right]+\tilde{c}\dot{\hat{c}}=-s\varepsilon \varphi \left(s\right)-\tilde{c}s\dot{e}+\tilde{c}\dot{\hat{c}}$$

In Equation (24), $0\leq s\varphi \left(s\right)\leq s\;sgn\left(s\right)=\left|s\right|$, then $-s\varepsilon \varphi \left(s\right)\leq 0$, and the equality sign holds when and only when *s* = 0. Let $-\tilde{c}s\dot{e}+\tilde{c}\dot{\hat{c}}=0$, yielding $\dot{\hat{c}}=s\dot{e}$, and increasing both the gain coefficient *γ_c_* and the initial value *k_c_*_0_; the adaptive law for the parameter *c* is:

(25) $$\hat{c}=\gamma_{c}\int s\dot{e}dt+k_{c_{0}}$$

Prove the stability of the system based on the second method of Lyapunov stability theory:

(1) The *V* function represented by Equation (23) is positive definite;
(2) Substituting Equation (25) into (24) gives $\dot{V\leq 0}$, and the equality sign holds only at s=0 and $\tilde{c}=0$.

Therefore, the system is located at the origin, and the following error at zero is asymptotically stable.

The parameter *c* was adaptively estimated and applied to the simulation model, defining the initial value of the switching gain *k_ε_*_0_ = 230, the boundary thickness *k_a_*_0_ = 50, the adaptive gain *γ_ε_* = 500, *γ_a_* = 10. The initial value of the convergence parameter *c* is *k_c_*_0_ = 30, the adaptive gain factor *γ_c_* = 3000, the input signal is constant, and the simulation runs for 12 s.

**Figure 8:**
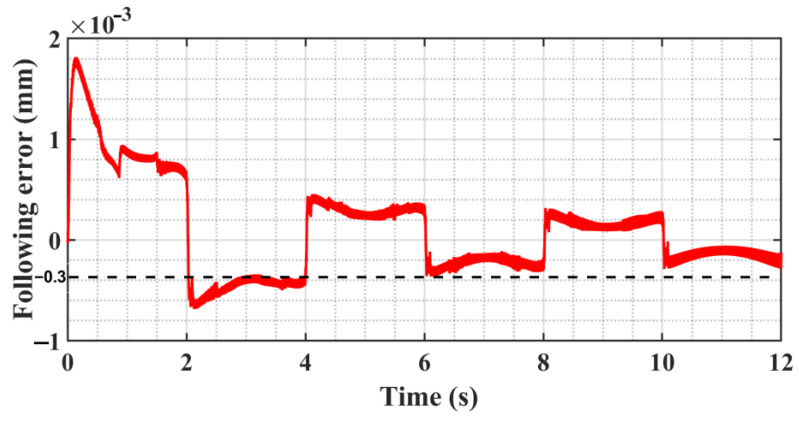
Following error curve.

Figure 8 simulation outcomes indicate that after the adaptive estimation of the parameter *c*, the system can spontaneously improve the convergence of the algorithm and further reduce the “static difference”; finally, the following error is reduced to about 0.3 μm, but there is still a small “static difference” error. In this case, depending on the algorithm, convergence alone cannot achieve a smaller following error, and if *c* is forced to take too large a value, it will cause a strong vibration of the system. The above problems are analyzed and solved in the experimental session.

## 5. Experimental Verification

### 5.1. Experimental Platform

**Figure 9:**
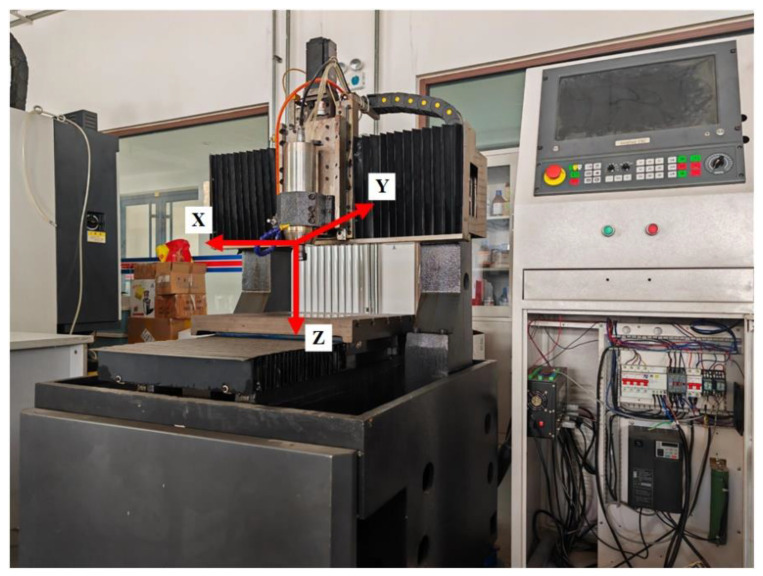
Three-axis CNC engraving and milling machine experiment platform.

This experiment is based on a three-axis CNC engraving and milling machine platform, as shown in Figure 9; the controller adopts digital amount to execute the servo control algorithm (1 mm = 2000 cts), and the driver adopts the torque control mode, which constitutes a closed-loop control system and ensures that the machine still maintains stable motion and positioning accuracy under the situation of load change.

### 5.2. SMC of Trajectory Motion Experiments

**Figure 10:**
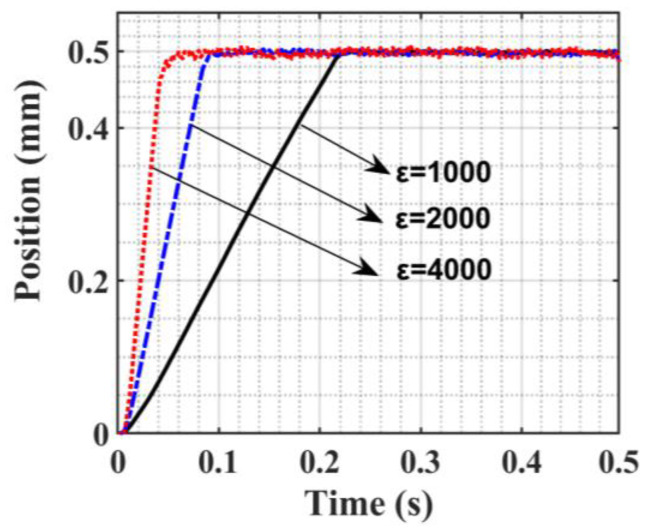
Step position response curve.

**Figure 11:**
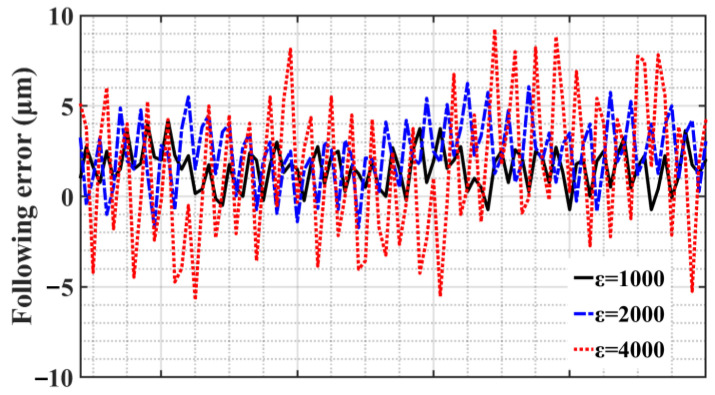
Steady state following error of step position response curve.

According to the relevant characteristics of SMC chatter, the 0.5 mm position step signal is used as the input signal for experimental verification. The SMC is parameter *c* = 500. Figure 10 shows the step position response curve when switching gain *ε* takes different values. Since the SMC law adopts an equal-velocity convergence law, each curve shows a convergence process of reaching the steady state at a constant rate. As the switching gain *ε* increases, the time to reach the peak time is shortened accordingly, which is 0.23 s, 0.09 s, and 0.05 s, respectively, and the convergence rate is increased accordingly. The following error curve presented in Figure 11 below shows that when a steady state is reached, chatter intensifies accordingly.

**Figure 12:**
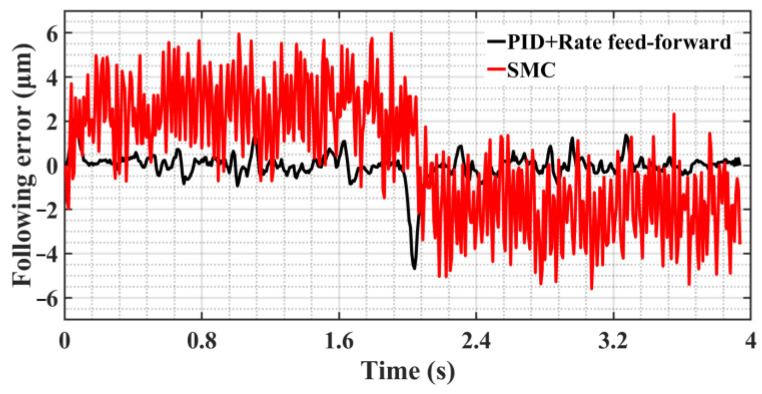
Comparison diagram of following error curve of two control methods.

The parabolic position signal $r=-cos\left(0.5\pi t\right)+1\mathrm{mm}$ is used as the test command for the machining experiment, and the parameters of the SMC are set to *c* = 500, *ε* = 1500. Based on the following error shown in Figure 12, the analysis shows that SMC can eliminate the disturbance of various nonlinear factors under the condition of satisfying disturbance matching, but it produces excessive chattering, which leads to a decrease in control performance, the increase in motor energy consumption, and the reduction in the service life of the CNC machine tool, and the disadvantages outweigh the advantages in general.

### 5.3. ASMC of Trajectory Motion Experiments

The parabolic position signal $r=-cos\left(0.5\pi t\right)+1\mathrm{mm}$ is used as the test command for the machining experiments, which are carried out on the CNC machine based on different control methods (including SMC, SMC+MRAC, and ASMC+MRAC), and the following error generated by the three control methods are compared. Table 3 displays the control parameters for different control methods.

**Figure 13:**
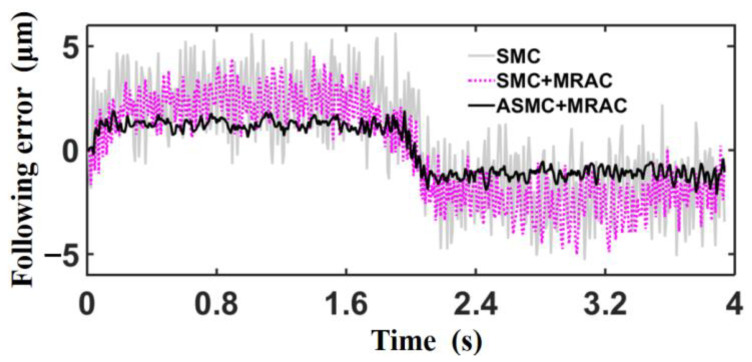
Comparison diagram of following error curve of three control methods.

Figure 13 shows the following error curves under the three control methods. The analysis shows that, by comparing the two control strategies of SMC and SMC+MRAC, adding MRAC in the control inner loop can effectively eliminate the influence of nonlinear factors so that the switching gain *ε* of the SMC can satisfy the disturbance matching condition with a smaller value, thus reducing the chattering and improving the control performance. Compared with SMC+MRAC, ASMC+SMC can adaptively adjust the control parameters, which makes it easier to obtain the optimal control parameters and further reduces the following error and chattering. Table 4 shows the variance in the following error data shown in Figure 13. With the continuous optimization of the control algorithm, the deviation of the following error decreases gradually, which indicates that the amplitude of chatter of the SMC decreases gradually.

The following error under ASMC+MRAC control in Figure 13 shows that after the control system is stabilized, the error is always stable within 2 μm, and there is a “static difference” phenomenon, which is due to the control algorithm containing a number of adaptive algorithms; adaptive algorithms have a hysteresis effect, and the hysteresis effect is more obvious when the input signal is time-varying. On the other hand, there is sliding friction resistance in the mechanical transmission link of CNC machine tools, and the direction of sliding friction resistance is opposite to the direction of speed, and the control system makes it difficult to achieve better convergence under the combined effect of the two. The previous part of the study improved the convergence of the algorithm by adaptive estimation of the parameter *c*, reducing part of the “static difference”, which at this point is mainly caused by the sliding friction resistance and is in the opposite direction of the velocity direction, causing the actual position output signal to always lag behind the desired input signal.

**Figure 14:**
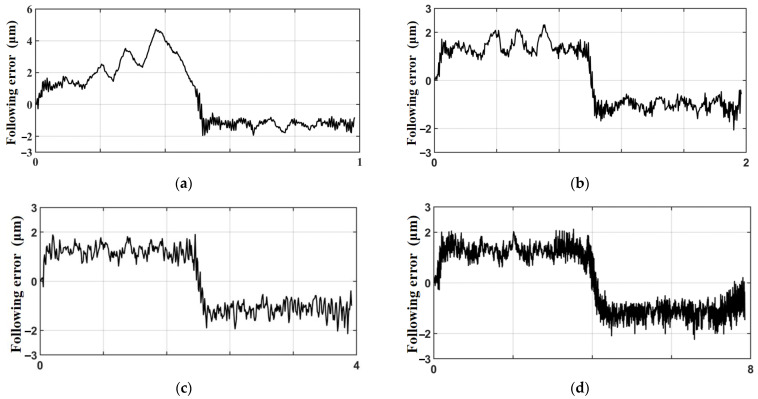
Following error graphs at different running times: (**a**) running time 1 s; (**b**) running time 2 s; (**c**) running time 4 s; (**d**) running time 8 s.

In order to verify the relationship between the “static difference” and the feed rate, the same trajectories were completed at machining times of 1 s, 2 s, 4 s, and 8 s, respectively, under the control method of ASMC+MRAC. Figure 14 shows the following error curves under each machining time, and the analysis shows that the following error of the trajectory is mostly stable at about ±2 μm, and the overall value is not much different (when the running time is 1 s, due to the relatively fast running speed, there is an obvious adaptive process in the first half of the trajectory, and the second half of the trajectory still tends to be stable).

**Figure 15:**
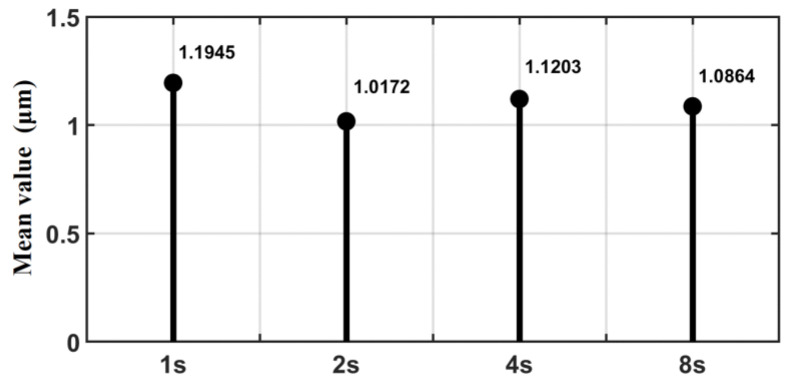
Mean value of following error for each processing run time.

Figure 15 presents the average value of the absolute value of the following error at different processing times (the data in the second half of the error curve). The analysis shows that there is no positive or negative correlation between the mean size of the “static difference” and the operating speeds, which further indicates that after enhancing the convergence of the algorithm, the sliding friction resistance is relatively fixed state and does not change with the speed, and the direction of resistance is opposite to the direction of motion.

**Figure 16:**
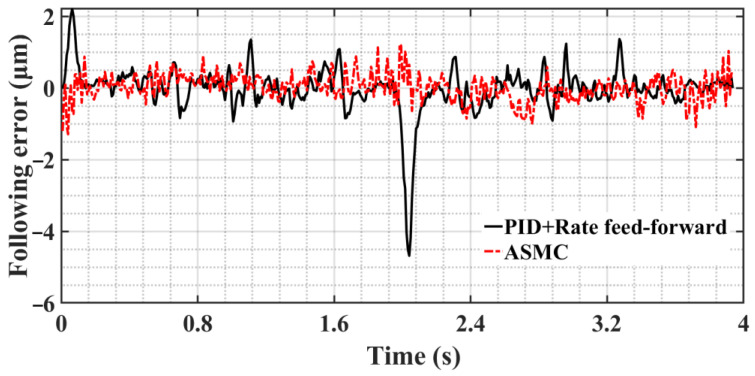
Comparison diagram of following error curve of two control methods.

For the “static difference”, which has a definite value, a position feed-forward compensation of 2 μm is added to the input desired signal, and the direction of the position compensation is the same as the direction of the velocity so that the hysteresis effect can be eliminated on the following error. Figure 16 shows the following error curve after adding position feed-forward compensation; the control method is ASMC+MRAC and compared with the following error curve under PID+rate feed-forward control. In the stage of static friction to sliding friction, the maximum following error decreases from 2.21 μm to 1.29 μm, a decrease of 41.6%; in the speed direction change phase, the maximum following error decreases from 4.68 μm to 1.23 μm, a decrease of 73.7%. The analysis shows that the introduction of position feed-forward compensation can offset the influence of sliding friction resistance, and the ASMC+MRAC proposed in this paper, compared with the PID+rate feed-forward control, effectively eliminates the phenomena of “Zero crossing” and “Over-quadrant sharp corners” caused by nonlinear friction during the initial response and commutation process of the system, and the following error is a substantial reduction.

**Figure 17:**
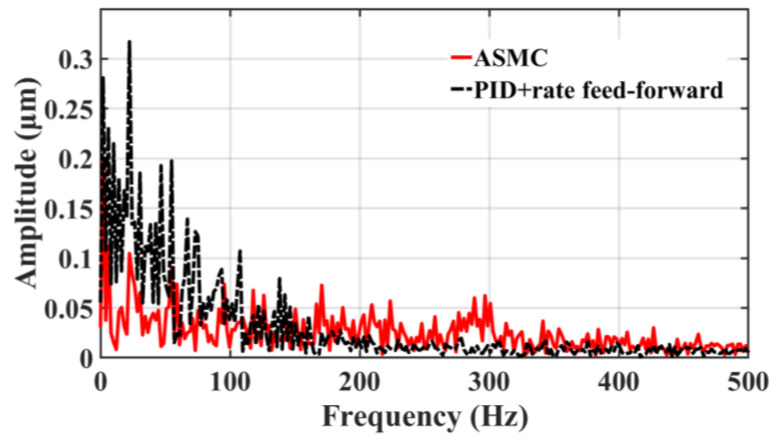
Comparison between ASMC and PID+rate feed-forward spectrum analysis.

**Figure 18:**
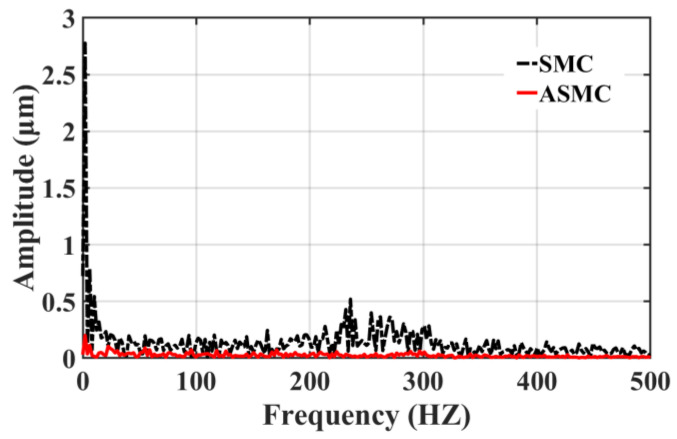
Comparison between SMC and ASMC spectrum analysis.

Figure 17 and Figure 18 clearly illustrate the following error spectrum analysis of the two control methods. The analysis shows that in the relatively low-frequency range of 0–150 HZ, the error amplitude of ASMC is lower than that of the traditional PID+rate feed-forward control, which effectively inhibits the mechanical resonance in this range, noise, and other disturbances, improving the stability of the system; in the high frequency range of 150 to 340 HZ, the amplitude of the ASMC is slightly larger than that of the PID+rate feed-forward control due to the inevitable chatter of the SMC itself. As shown in Figure 18, compared with the traditional SMC, the ASMC method designed in this paper substantially reduces the overall chattering, the linearity error at the low frequency is greatly reduced, the chattering at the high frequency is greatly weakened, and the maximum chattering amplitude is lowered from 0.52 μm to 0.057 μm, which is a reduction of 89.02%, and the control system’s stability has been significantly enhanced.

## 6. Conclusions

This paper introduces an ASMC strategy that is based on MRAC as its approach. The control method effectively reduces the influence of chattering generated by SMC, which reduces the influence of system nonlinear friction and time-varying factors on the control accuracy and can adaptively estimate the control parameters according to the changes in the control system, thus solving the problem of relatively cumbersome manual debugging parameters.

First, a feed servo mechanism system containing the LuGre friction model is established in the control inner loop, and the MRAC is used to improve the controlled object “invariance” so that SMC satisfies the disturbance matching and reduces the chattering effect under the condition of smaller switching gain. The control outer loop used ASMC to replace the sign function with a continuously smooth bipolar function to radically smooth the output signal and used adaptive optimization to determine the appropriate switching gain and boundary thickness control parameters. To address the issue of inadequate convergence of the control algorithm, the *c* value in the sliding surface is adaptively estimated, and the influence of “static difference” is further eliminated by using position feed-forward compensation. The simulation results substantiate the efficacy of the proposed approach, while the experimental findings demonstrate the practical viability of the method.

## Figures and Tables

**Table 1:** Equivalent double inertia mechanical model parameters.

Symbols	Name	Values
*J*_1_	Inertia of motor	2.85 × 10^−4^ kg·m^2^
*J*_2_	Inertia of load	5.12 × 10^−5^ kg·m^2^
*K*	Equivalent stiffness	18.29 N·m/rad
*C*	Equivalent damping	0.064 N·m/rad

**Table 2:** LuGre friction model parameters.

Symbols	Name	Values
*F_s_*	Max static friction	0.04263 N/m
*F_c_*	Coulomb friction	0.0091 N/m
*V_s_*	Stribeck velocity	0.007353 m/s
*σ_0_*	Stiffness coefficient	8.0274 N/m
*σ*_1_	Damping coefficien	2.343 N·s/m^2^
*σ*_2_	Viscosity coefficient	0.02772 N·s/m^2^

**Table 3:** Control parameters of the three control methods.

Control Methods	Control Parameters
SMC	*c* = 500
	*ε* = 1500
SMC+MRAC	*c* = 500
	*ε* = 500
ASMC+MRAC	*γ_ε_* = 500 *k_ε_*_0_ = 230
	*γ_a_* = 10 *k_a_*_0_ = 50
	*γ_c_* = 3000 *k_c_*_0_ = 250

**Table 4:** The variance of the following error.

Control Methods	Variance
SMC	6.88
SMC+MRAC	5.76
ASMC+MRAC	1.21

## Data Availability

Data are contained within the article.
